# Current Applications and Future Prospects of High-Throughput *Omics* Technologies in Animal Reproductive Biology

**DOI:** 10.3390/ani16152367

**Published:** 2026-08-03

**Authors:** Tlou C. Kujoana, Nthabiseng A. Sebola

**Affiliations:** Department of Agriculture and Animal Health, College of Agriculture and Environmental Sciences, University of South Africa, Florida 1709, South Africa; kujoatc@unisa.ac.za

**Keywords:** animal reproduction, animal biotechnology, reproductive biology, bioinformatics

## Abstract

Recent developments and applications of high-throughput *omics* technologies have modernised animal biotechnology, offering extraordinary comprehension of animal reproduction. The current review discusses the applications and advancement of high-throughput *omics* technologies in animal reproductive biology. The data for this review were sourced from old and recently published work in different journals. The internet databases used to access the manuscripts were ResearchGate, Google Scholar, ScienceDirect, Web of Science, PubMed, and the Directory of Open Access Journals. The results showed that *omics* technologies have been previously used in human biology, and with time, have come to be introduced in animal reproductive biology. Studies using these technologies showed that genomics analysis identified at least 180 CNVs and 49 candidate genes enriched in vital fertility pathways, including retinol metabolism, steroid hormone biosynthesis, and Hippo signalling, while transcriptomics analysis screened the molecular dynamics of reproductive indicators at both the cellular and molecular levels. Pig oocytes matured in vitro or in vivo were subjected to transcriptome analysis. These analyses generate large amounts of ‘Big Data’. As a result, *omics* can handle large amounts of data generated through the analyses, but it needs bioinformatics experts and advanced computers, making it less affordable at the farm level. However, widely utilised technologies, including assisted reproductive technologies (ARTs), have hit their limit after years of use. To break this plateau of traditional ARTs, future research must give integrative ARTs and high-throughput *omics* technologies top priority, as they have clearly been shown to improve farm animals’ reproductive success. The synergy of increased reproductive efficiency and a smaller ecological footprint is crucial for improving the accuracy and sustainability of livestock systems, ultimately promoting global food security.

## Simple Summary

In animal reproductive biology, high-throughput *omics* technologies allow researchers to study hundreds of molecules and massive amounts of data at once. These tools offer a comprehensive understanding of the cellular processes involved in animal reproduction. By offering a profound molecular-level understanding of fertility, gamete quality, and embryonic development, *omics* technologies, including genomics, transcriptomics, proteomics, and metabolomics, are revolutionising animal reproduction. These techniques advance breeding from traditional phenotypic observation to accurate, data-driven selection by examining large datasets. Their integration with assisted reproductive technologies into animal biotechnology offers the potential to significantly enhance reproductive outputs in farm animal production through the provision of a deeper understanding of the molecular physiological mechanisms behind fertility.

## 1. Introduction

The substantial production of livestock contributes significantly to the global agricultural sector by providing vital animal proteins that enhance food security. This can be achieved through the enhanced reproductive efficiency of farm animals, as it is fundamental to sustaining their maximal productivity [1,2]. High production of farm animals enhances the production and accessibility of animal proteins and increases sales of live animals, fur, hides, and organic matter, to name a few by-products. This therefore aids in combating poverty, unemployment, hunger, and nutrition security, which is in line with the Sustainable Development Goals agenda 2030 and the South African National Development Plan 2030 [3,4]. Hence, farm animals’ reproductive efficiency is vital to their optimal production and to sustaining global agricultural growth, which was previously accomplished through the successful use of assisted reproductive technologies (ARTs) [5]. The success of these ARTs has previously relied on precise phenotypic selection, basic genetic methods, gamete selection, embryo selection, and the determination of the best endometrial window for embryo implantation [6]. Despite the critical need for increased animal productivity, common ARTs have reached a pinnacle of success after being employed for years to assess the key reproductive processes and can no longer address the biological complexities of animal reproduction, including multiple pregnancy risk and species-specific mechanisms [7,8,9]. Therefore, the adoption of more reliable, efficient, and sustainable criteria is imperative to successfully enhance the reproductive outputs and increase animal productivity. Because understanding reproductive processes in farm animals is critical for improving fertility, welfare, and food security globally. The emergence of high-throughput *omics* technologies created new avenues for improving animal reproductive efficiency and comprehending the molecular basis of animal reproduction [10]. This includes proteomics, metabolomics, transcriptomics, genomics, and their interaction with ARTs.

High-throughput *omics* technologies possess specific tools that can be used to identify genes associated with reproductive parameters, such as fertility, and are able to identify the patterns of gene expression at numerous stages of reproduction and offer insight into locating proteins involved in reproductive processes [11]. These tools can also assist in an inclusive approach to the molecular and cellular mechanisms that correspond to key characteristics, in addition to identifying genes and the metabolic pathways essential for fertility [12]. Therefore, the ability to carry huge datasets at multiple biological levels provides unique insights into complex collaborations amongst genes, proteins, and metabolites [13]. This integrated approach, often called systems biology, will overcome current reproductive plateaus, thereby optimising livestock breeding programmes, enhancing animal welfare, and improving the permanence of livestock production systems. Hence, high-throughput *omics* technologies should be ideally objective, accurate, fast, and affordable, especially at the farm level. Although high-throughput *omics* technologies have the potential to improve cattle breeding, there is a great deal of fragmentation in the existing studies. There is a significant knowledge gap about the practical integration of proteomics, metabolomics, transcriptomics, and genomes with conventional ARTs. Moreover, there is no comprehensive framework outlining the obstacles at the field level, financial constraints, and species-specific restrictions that keep these cutting-edge molecular techniques from being applied at the farm level to overcome the existing reproductive plateaus. Hence, this review seeks to discuss the following: (1) the historically development of high-throughput technologies; (2) the description and application of *omics* technologies in farm animals’ reproduction; (3) the interaction of high-throughput *omics* technologies with ARTs; (4) the challenges associated with the use of *omics* technologies in livestock reproduction; and (5) prospects for the continuous use of high-throughput *omics* technologies in animal reproduction.

## 2. Materials and Methods

### 2.1. Literature Search

The goal of the literature search was to find contemporary peer-reviewed and online articles about the history, development, and use of high-throughput *omics* technologies to improve farm animal reproduction. The internet databases used to access the manuscripts were ResearchGate, Google Scholar, ScienceDirect, Web of Science, PubMed, and the Directory of Open Access Journals. The following keywords were used in the search process: “high-throughput technology”, “*omics* technologies”, “*omics*”, “genomics”, “transcriptomics”, “proteomics”, “metabolomics”, “reproduction”, “fertility”, and “farm animals”. Furthermore, other pertinent data, papers, or publications were found using the references provided in the articles.

### 2.2. Selection Criteria (Inclusion and Exclusion)

**Figure 1:**
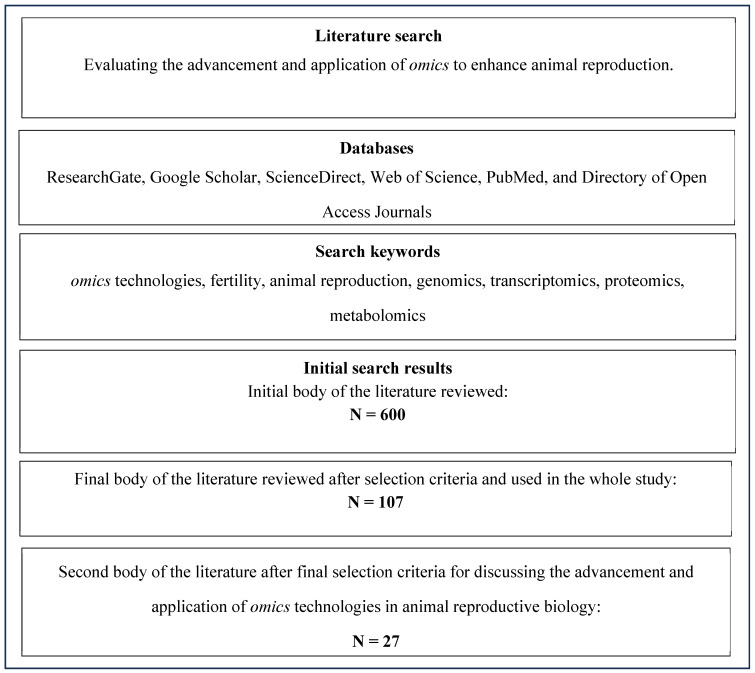
The literature search strategy and selection criteria for studies that specifically focused on the use of *omics* technologies to enhance animal reproduction.

The searched studies were categorised following the method described by Sjofjan et al. [14]: written consistently in English; reporting on the advancement of the application and advancement of high-throughput *omics* technologies in animal reproductive biology. Criteria for the study to be included in the database were as follows: (1) it is available as an open full text, abstract, or poster; (2) the piece is published in a book or peer-reviewed journal; (3) the study or a portion of it discusses the evolution of high-throughput technologies historically; (4) the topic aligns with our main focus; (5) the study or a portion of it discusses the development and use of high-throughput technologies in animal reproduction; (6) the article or a portion of it discusses genomics, transcriptomics, proteomics, and metabolomics; (7) the article or a portion of it discusses the integration of *omics* and assisted reproductive technologies and the difficulties in applying *omics* technologies to animal reproduction; (8) the article was published between 2010 and 2015 or 2016 and 2025, as these are the periods when the use of high-throughput *omics* technologies became popular. These search criteria produced about 600 relevant studies. The full texts and abstracts of these articles were also examined, evaluated, and categorised in accordance with the inclusion criteria. The final database consisted of 107 articles used in the whole study. Furthermore, the secondary selection criteria were employed, which resulted in 27 articles that are specifically focused on the 4 high-throughput *omics* technologies applied in animal reproductive biology. The literature search methodology employed for this work is summarised in Figure 1, below.

### 2.3. Sorting of Selected Relevant Articles

The chosen articles were sorted based on the high-throughput *omics* technologies they addressed and the year of publication (Table 1). Following the analysis of the selected articles, data on recognised and widely used high-throughput *omics* technology in animal reproduction biology was assessed. Most publications on high-throughput *omics* technologies concentrate on the development and use of *omics* in human reproduction, animal breeding, and genetics; the identification and selection of genes linked to growth, health, and adaptability; and the analysis of massive amounts of data produced. However, the development and use of high-throughput *omics* technologies in farm animal reproductive biology receives less attention. As a result, not much has been written about this subject to date.

## 3. Search Results and Discussion

### 3.1. Historical Development of High-Throughput Omics Technologies

**Figure 2:**
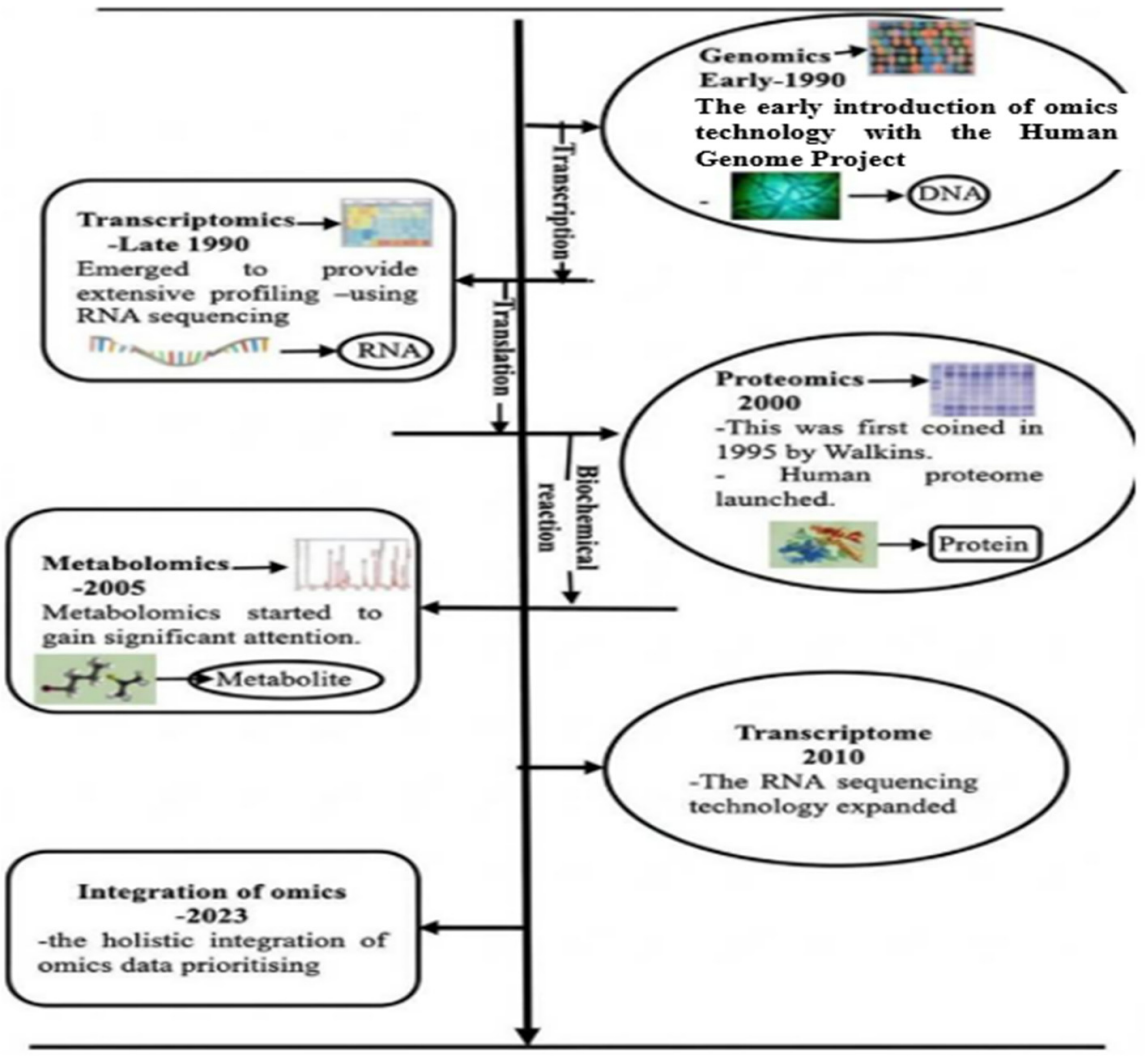
The diagram shows the historical evolution and development of high-throughput *omics* technologies.

Historically, high-throughput *omics* technologies were developed in the latter half of the 20th century, propelled by advances in mass spectrometry and DNA sequencing technologies. The advancements led to comprehensive, in-depth analyses of biological substances that revolutionised the life sciences and personalised medicine, replacing targeted gene studies [15]. The suffix “*omics*” stands for the groundbreaking technological development in the last thirty years that allows us to analyse thousands of molecules at once. The *omics* technologies were first introduced in the Human Genome Project (HGP) between 1990 and 2003 (Figure 2), with its official launch in 1990 spurring important scientific breakthroughs. Since the HGP first employed the time-consuming Sanger sequencing, its completion in 2003 signalled the beginning of a new era in biomedical science by providing access to extensive genomic data [16,17]. However, this technique was time-consuming and expensive.

The development of other *omics* technologies began in the early 1990s and continues to this day. Transcriptomics emerged in the late 1990s using Sanger sequencing and microarrays; however, the first technique to identify RNA in tissue samples was reported to be in situ hybridisation in the late 1960s, which laid the groundwork for spatial transcriptomics. Around the year 2008, RNA sequencing (RNA-Seq) was developed to provide extensive profiling, where high-throughput sequencing of entire transcripts began, providing unprecedented resolution of splice variants and non-coding RNA [18]. In 2020, spatial transcriptomics was named method of the year by Nature Methods. This preserves the spatial context of gene expression within tissues [19]. Proteomics technology involves the large-scale study of proteins that are functional products of genes. In the late 1980s, John Fenn and Koichi Tanaka developed the Soft Ionisation method to ionise large proteins without breaking them, making mass spectrometry (MS) feasible for biological samples. In 1975, 2D Gel Electrophoresis was used for the first time, successfully enabling the separation of proteins by charge and mass. In 1994, Marc Wilkins identified and quantified high-throughput proteins using MS and nuclear magnetic resonance spectrometry (NMR), and the enhanced MS and NMR spectrometry methods were advantageous for metabolomics [20,21]. The phrase “metabolic profile” was first used in 1971 when Horning and colleagues integrated gas chromatography–mass spectrometry (GC-MS) to profile human urine. Moreover, in the early 2000s, companies like Illumina and Roche developed and introduced massively parallel sequencing, such as next-generation sequencing technologies, which greatly reduced the cost and time of DNA sequencing, leading to improvements in this field [18]. Larger-scale research was therefore made possible, including studies of the animal genome that go beyond the human genome.

Generally, the evolution of high-throughput *omics* technologies has been marked by an ongoing pursuit of higher throughput, reduced costs, and enhanced resolution, progressing from the examination of individual genes or proteins to a comprehensive understanding of complete biological systems [22]. Figure 2, below, presents the historical evolution, development, and application of *omics* technologies.

### 3.2. Description and Application of Omics Technologies in Animal Reproduction

**Figure 3:**
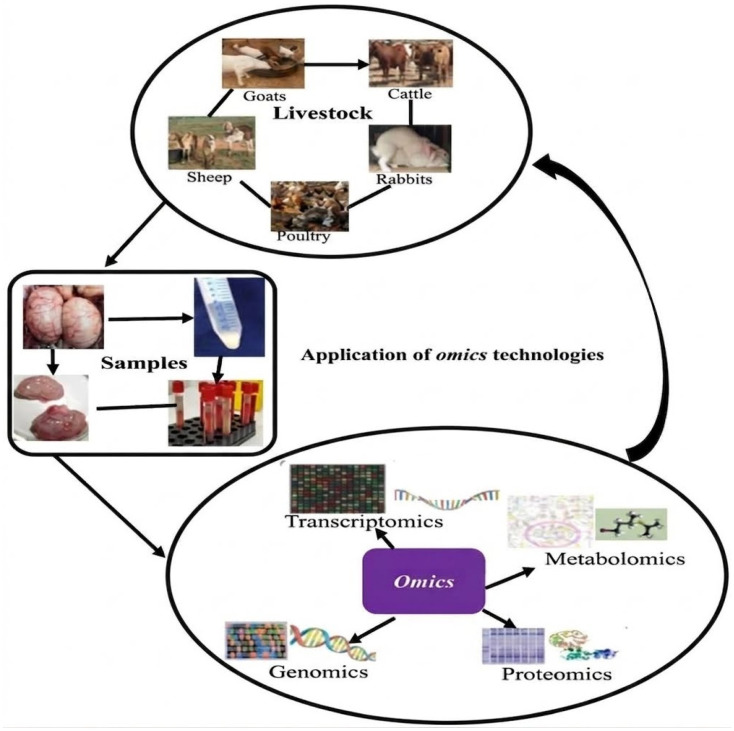
An illustration of how *omics* technologies (genomics, transcriptomics, proteomics, and metabolomics) are applied to farm animals’ reproductive biology.

High-throughput *omics* technologies are modernised tools that were developed to collect and analyse high-throughput data on proteins (proteomics), mRNA transcripts (transcriptomics), gene sequences (genomics), and metabolic profiles (metabolomics) of a given cell, tissue, organ, or whole organism at a specific time [15], as shown in Figure 3. They are thought of as extensive research instruments that allow scientists to investigate intricate biological processes at the molecular level, offering valuable information on the reproductive physiology and pathology [23,24]. The information from the *omics* data can be integrated through robust bioinformatics and computational tools to the systems biology level. Their collective use in advancing animal reproduction provides a gap bridge between the animal’s potential (genotype) and its actual reproductive performance (phenotype) [25].

Primarily, genetic selection of farm animals for breeding purposes relied on their phenotypic traits, including production characteristics and breeding value estimations [26]. With time, traits such as reproduction and longevity traits, animal health, stress tolerance, disease resistance, and animal welfare traits became important characteristics of genetic improvement programmes [27]. The application of these technologies improved the general understanding of animal reproductive efficiency and further revolutionised animal breeding by providing accurate molecular insights by going beyond conventional phenotypic selection [28]. A study by Menon et al. [29] discussed how *omics* technologies could be utilised in identifying the genetic variants associated with desirable reproductive traits and biomarkers for reproductive disorders, enabling more effective genetic selection and early diagnosis and intervention, respectively. Generally, new developments in *omics* technology have enhanced our knowledge of farm animal reproduction by providing detailed descriptions of deep molecular insights, exposing new biological information, and speeding up the discovery of genetic and regulatory variables that affect farm animal reproductive traits [30].

#### 3.2.1. Genomics

An organism’s complete genetic material, or genome, is the focus of the multidisciplinary field of biology known as genomics [31]. This is a key aspect of modern animal breeding that involves identifying and characterising all genes, non-coding DNA regions, and how these elements interact with each other and with the environment to influence the traits and functions of an organism [32]. By analysing the relevant genome, genomics techniques are mainly used to study variations between individuals at the germline and somatic levels. This includes the development of DNA microarray technology [33], first-generation Sanger sequencing [34], next-generation sequencing (NGS), or second-generation massively parallel sequencing, and lastly, the third-generation long-read sequencing (TSG) [35]. Furthermore, amongst genomics techniques, NGS is the one that provides the strongest diagnostic and breeding capabilities. However, this technique is said to be the most expensive due to the high, multi-layered cost of establishing and maintaining infrastructure, which is intensified by hidden logistical expenses, rather than just the cost per reaction. This limits its use on the farm level in most developing countries [27,32].

The use of genomics technology in animal reproduction enables breeders to make more precise, targeted, and efficient selection decisions. This technology allows for the improvement of fertility and other complex variables related to reproduction that were previously difficult to measure and had low heritability [36]. One of the primary applications of genomics in enhancing animal reproduction is genomic selection, which is particularly beneficial because it can be used to select animals for breeding early in life—often just after birth—by analysing thousands of genetic markers, such as single-nucleotide polymorphisms (SNPS), across the entire genome, without needing reference to their own breeding or production records [37]. Recently, there has been increased focus on identifying the genetic variations associated with litter size in animals, especially in goats. For example, genome-wide association studies have identified an SNP variant linked to litter size at birth; *GABRA5*, *AKAP13*, and *SV2 B* are among the potential genes annotated from these variants [38]. However, focusing solely on SNPs does not provide a comprehensive understanding of their genetic basis. Therefore, copy number variations (CNVs) are a significant form of structural variation involving changes in the number of large genomic segments, as they can significantly influence gene expression and function. Increasing evidence shows that CNVs contribute significantly to animal reproduction. A study by Zhao et al. [39] examined CNVs in Guizhou Black goats with varying litter sizes. They identified 180 CNVs and 49 candidate genes enriched in vital fertility pathways, including retinol metabolism, steroid hormone biosynthesis, and Hippo signalling. This indicates that CNVs may serve as valuable genomic markers for selective breeding aimed at increasing goat prolificacy. In a similar vein, Oliveira et al. [40] demonstrated that a CNV-based genome-wide association analysis in Holstein cattle found two CNVRs associated with three reproductive traits: none return rate, interval from service to conception, and calf survival rate.

**Figure 4:**
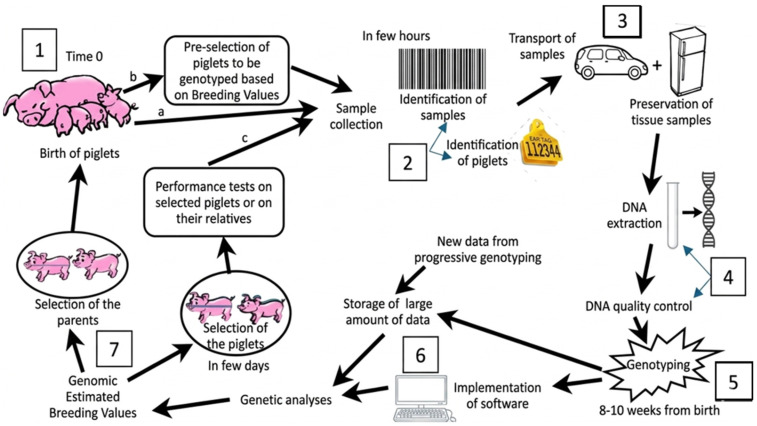
Diagram illustrating the useful elements of using genomic selection programmes to pigs: (1) collecting samples from (a) newly born piglets, (b) preselected piglets, or (c) pigs in the performance test station; (2) identifying piglets and samples using bar coding systems; (3) transporting and preserving samples; (4) laboratory analyses, including DNA extraction and quality controls; (5) genotyping; (6) storing the vast amount of genomic data, anticipating the increasing amount of data derived by the continuous genotyping in the population over time; (7) using genomic breeding value (Source: [41]).

Additionally, genomics can be integrated with assisted reproductive technologies, such as in vitro fertilisation, multiple ovulations, artificial insemination, and embryo transfer. This integration increases the precision of selection and intensifies the use of exceptional animals [37]. Thus, further study on this subject is required. The practical aspects of applying genomic selection programmes in pigs are shown in Figure 4, below. This programme used DNA data to predict which piglets will have the best traits, allowing farmers to make breeding decisions much faster than traditional methods. At the start of the cycle, piglets are born, and then pre-selection is used to determine which piglets will be tested based on the known breeding values of their parents. Tissue samples like ear notches are then collected from the selected piglets, and to be accurate, both the piglet and the sample are uniquely identified. To prevent DNA deterioration prior to formal analysis, samples are then transported using a cold chain to a laboratory for preservation. Additionally, the DNA is extracted from the tissue sample and sent to quality control to ensure that the genetic material is clean enough to yield reliable results. At roughly 8–10 months after birth, the pig’s DNA is scanned to identify specific genetic markers to provide a genetic map of the individual piglet. As a result, the piglet’s potential is determined by comparing its markers to a reference population using sophisticated software and algorithms to interpret the collected data, which is referred to as Big Data. The final product, known as the Genomic Estimated Breeding Value (GEBV), then forecasts the pig’s performance and the quality of its offspring. The best pigs are chosen to be parents for the following generation based on these values.

#### 3.2.2. Transcriptomics

Transcriptomics technology is typically used to analyse the complete RNA sequence (RNA-seq) of an organism to find genes that are activated in specific reproductive characters. This refers to the analysis of reproductive tissues like ovaries, testes, and uterus provided during key stages such as oocyte maturation, spermatogenesis, and embryo implantation through RNA sequencing [42]. Moreover, transcriptomics further studies how long non-coding RNAs and regulatory non-coding RNAs, such as microRNA, affect gene expression and are connected to reproductive function [43]. As a result, it has been discussed that RNA-seq has revolutionised the analysis of transcriptomes. Its primary benefits include a wide dynamic range and sensitivity, accurate and objective transcript quantification, and thorough coverage of all expressed sequences in a particular tissue sample [44]. This information facilitates comprehension of the regulatory systems that control fertility and reproductive function.

**Figure 5:**
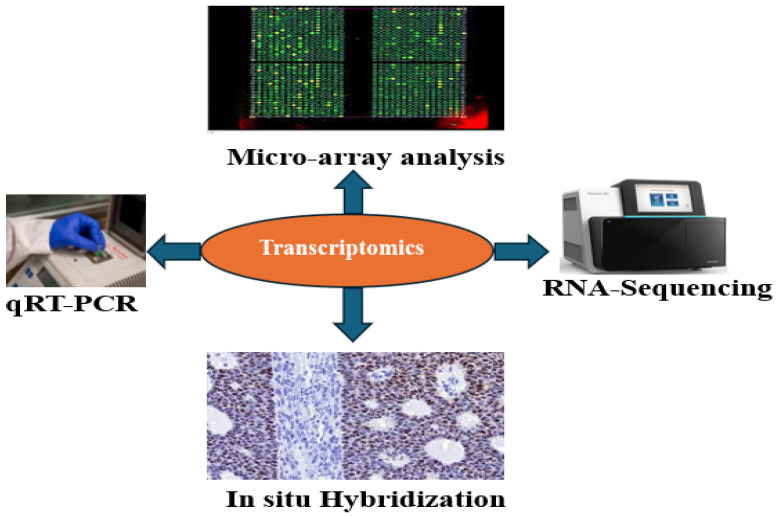
Depicts various methods used during the transcriptomic analysis (source: [10]).

Transcriptomics has used a variety of methods (Figure 5) to analyse patterns of unique gene expressions in animal reproduction. These methods enable researchers to identify genes with distinct expression patterns as well as the signalling pathways and regulatory networks involved in reproductive processes [45]. RNA-seq, in situ hybridization (ISH), quantitative real-time polymerase chain reaction (qRT-PCR), and microarray analysis are often-employed techniques in transcriptomics for animal reproduction [46]. The RNA-seq method has been employed in extensive scientific studies on many farm animals up to this point, replacing the Maxam and Gilbert chemical degradation sequencing approach [47]. It is a highly successful technology that makes it feasible to sequence and quantify the quantity of each RNA molecule in a sample and can detect a substantially larger percentage of differentially expressed genes, particularly rare or low-abundance transcripts [48]. A study conducted by Xiong et al. [49] focused on the regulatory function of non-coding RNAs (ncRNAs) in ovarian atresia linked to broodiness in hens, a trait that is harmful to egg production. Thus, they employed whole-transcriptome sequencing and discovered hundreds of circular RNAs (circRNAs), long noncoding RNAs (lncRNAs), and differently expressed microRNAs (miRNAs) that formed a network of competing endogenous RNAs. Genes (*THBS1* and *MYLK*) that are specifically controlled by miRNAs and circRNAs have been connected to ovarian function pathways such as hormone secretion, cytokine signalling, and intracellular matrix receptor connections. The qRT-PCR has the lowest detection and the broadest dynamic range and may accurately detect changes in genes that might be missed by microarrays. It exhibits greater resistance to partially degraded RNA samples than hybridization-based methods [50]. Microarray study employs probe hybridisation to identify known sequences and minor chromosomal deletions or duplications [51]. This method, which comprises DNA and protein microarrays, has been a mainstay of reproductive research since it provides high-throughput capabilities to investigate genetic variants and gene expression, such as in endometrial receptivity studies and the detection of CNVs during pregnancy. However, compared to more recent technologies, its application is constrained by its dependence on known sequences and lesser sensitivity for low-abundance genes [40,52]. Many transcriptomic studies have been carried out on livestock-related reproductive phenotypes, as shown in Table 2, below. According to Indriastuti et al. [52], a number of animal species, including bovines, horses, buffalo, pigs, goats, and poultry, have shown a correlation between the genes that produce fertility and semen.

Furthermore, through the integration of transcriptome and proteomic analysis of ovarian tissues obtained throughout breeding and non-breeding seasons, Shi et al. [53] assessed the seasonal reproductive physiology of dairy goats. Their research, which was supported by more than 1000 differentially expressed genes and more than 500 differentially expressed proteins, showed significant decreases in gonadotropin levels and follicular size during the non-breeding phase. Using steroid hormone production pathways, common genes such as *TMEM205*, *TM7SF2*, *SLC35G1*, *GSTM1*, and *ABHD6* were found to be putative mediators of inhibited follicular development. 

**Table 2:** The effect of application of different transcriptomic technologies on reproductive functions of different animal species.

Animal Species	Samples	Phenotypes	Techniques	Findings	References
Pig	Sperm	Embryo cleavage rate and sperm capacitation	qRT-PCR	While *MYC*, *CYP19*, *ADAM2*, *PRM1*, and *PRM2* genes were more important in the high cleavage group, *MYV* mRNA was reduced in the capacitated sperm.	[54]
Chicken	Sperm and testis	Sperm vs. testis	qRT-PCR and Microarray analysis	Microarray: 17,524 transcripts were found in sperm, of which 12.7% were found exclusively in sperm, and 83.7% were shared with the testis. Signal transduction (63.20%), embryonic development (56.67%), and cell structure (56.25%) were all attributed to upregulated transcripts.	[55]
	Sperm	Fresh vs. frozen sperm	Microarray analysis	In frozen sperm, 2115 genes showed differential expression, 2086 were downregulated, and 29 were upregulated.	[56]
Cattle	Sperm	High vs. low sperm fertility	RNA-sequencing	While 11,561 lncRNAs were found and 2517 were differentially expressed, 20,875 protein-encoding genes were found and 19 were differentially expressed.	[57]
		High vs. Sub-fertility	qRT-PCR	It has been established that CCDC174 is a predictor of bull fertility.	[58]
Buffalo	Sperm	High vs. low fertility	Microarray analysis	There were 51,282 transcripts identified, of which 4050 were DE and 709 were markedly dysregulated.	[59]
Horse	Sperm and testis	Sperm vs. testis	Microarray analysis and RNA-sequencing.	Microarray: 6596 of the 6761 gen/EST transcripts discovered in sperm and 11,112 in testis were shared. 19,257 sequence tags were mapped using RNA-seq. In testis, 12,013 transcripts were expressed.	[60]
Sheep	Testis	Germ ells vs. somatic cells	ScRNA-seq	A number of cell cycle, gamete production, protein processing, and mRNA surveillance pathways were enhanced in germ cells. Ribosome pathways were abundant in somatic cells. EZH2, SOX18, SCP2, PCNA, and PRKD are germ cell marker genes.	[61]

#### 3.2.3. Proteomics

**Figure 6:**
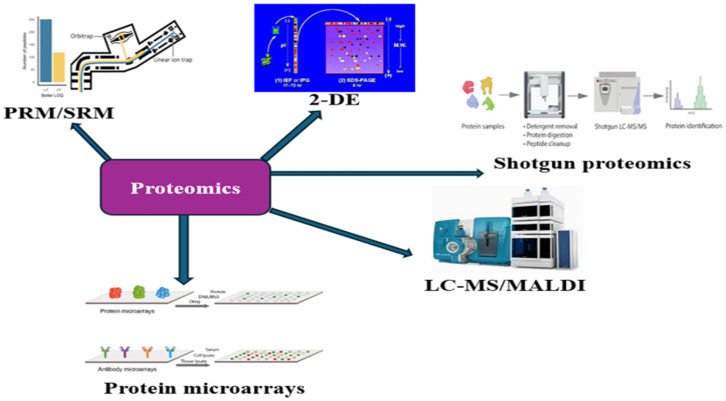
Various techniques that are commonly used during proteomic analysis (source: [10]).

Proteomics is one of the modern high-throughput *omics* technologies that was rapidly adopted and was first described by Wilkins and Williams in the mid-1990s [62]. This technology examines the information flow through protein signalling to determine the functional significance of all expressed proteins in a cell, tissue, or organism [63]. The discovery of protein signatures supporting reproductive physiology has been made possible by the development of proteomic technology. These signals provide a useful biomarker for evaluating and enhancing farm animal fertility [64]. Proteomic technology aims to use science and generate protein profiles and biomarkers that can be used to track, diagnose, and forecast animal pathology [65]. For instance, the infertility problem can be overcome by developing molecular ways to choose the best gametes and embryos as well as the most receptive endometrium, and by employing informative protein profiles linked to optimal reproductive output to predict fertility [66]. However, the application of proteomics in livestock research has historically been restricted due to its high cost and the lack of ideal protocols for different cell types in various species [67]. Nevertheless, the number of reports on proteomics studies in animal science is currently growing, leading to a better understanding of the health, productivity, and reproduction efficiency of animals, due to the development of new analytical techniques and computational tools, as presented in Figure 6, for the analysis of proteomic data [68,69]. Proteomics encompasses separation methods like two-dimensional (2D) PAGE, two-dimensional differential gel electrophoresis (2D-DIGE), one-dimensional sodium dodecyl–sulphate polyacrylamide gel (1D-SDS-PAGE), and liquid chromatography–mass spectrometry/mass spectrometry (LC-MS/MS) [70]. This technology has become the most advanced approach for predicting fertility with accuracy [71].

A sharp rise in the number of proteomics research linked to reproductive issues has been seen in the past years since the standardisation of mass spectrometry, building on genomics studies [72]. This technology has also enabled the identification of candidate protein markers of infertility for molecular breeding [73]. Moreover, proteomics can be used to better understand how proteins operate in male animals and how they affect sperm production, because male fertility is greatly influenced by sperm generation, maturation, and fertilisation capacity. Important proteins connected to these processes were found through proteomics studies, which offer crucial information on the molecular mechanisms behind effective male reproduction [74,75]. By studying the proteomes of animals such as chickens, rabbits, and the reproductive tissues of both small and large stocks, scientists can pinpoint proteins that are produced differently in fertile and infertile animals. These protein indicators can therefore be subsequently utilised to create diagnostic instruments that can precisely evaluate the fertility of breeding stock [76]. Since proteomics can now identify and measure thousands of proteins simultaneously, it offers a comprehensive view of the molecular changes taking place in animals’ reproductive systems [77].

Proteomics technology can be used to evaluate seminal plasma in addition to analysing reproductive organs. This is a fluid that surrounds and feeds the sperm in the male reproductive canal and contains a complex mixture of proteins that is essential for sperm motility, survival, and fertilisation [78]. Numerous studies have employed this method to investigate the effects of environmental factors on animal reproduction, including nutrition, stress, and diseases. Therefore, proteome profiling associated with reproductive tissues under certain conditions can identify protein markers linked to poor reproductive performance, which further offers important information about how external variables affect livestock fertility and reproductive success [79]. Studies by Swain and Pool [80,81], Hansen [82], Skakkebaek et al. [83], and Amann et al. [84] have examined male fertility defects, failed fertilisation in ARTs’, comparisons between male farm animals that are fertile and those that are infertile, and the development of diagnostic tools for infertile farm animals which further demonstrated the use of sperm protein profiling to identify potential biomarkers that may aid in the identification of sperm physiological impairment. In a study by Zhao et al. [68], eight fertility-related proteins were shown to be over-represented in Tibetan pigs with a heritable adaptation to a hypoxic environment using LC-MS/MS analysis of pig sperm. Another study demonstrated a consistent association between 1343 proteins and fertility based on LC-MS/MS analysis of seminal plasma proteins [83].

Therefore, using proteomics to find reproductive markers can assist in finding fertile male animals to lower non-return rates (NRR) and enhance productivity. Furthermore, identification of biomarkers may provide a mechanistic knowledge of the biological processes occurring at the cellular level during the embryo’s development before implantation [85]. Therefore, a comprehensive understanding of the embryonic proteome is essential since it should yield accurate markers of cellular metabolism and function during the preimplantation phase [86].

**Figure 7:**
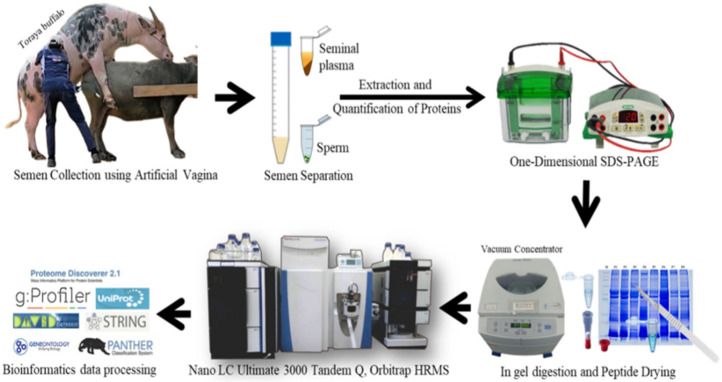
Application and workflow of proteomic analysis of Toraya buffalo seminal plasma using varied techniques. (Source: [89]).

Proteomic markers have also been used to model and assess reproductive stress scenarios. To profile serum proteins and oxidative damage producers, Liang et al. [87] created a hydrogen peroxide-induced oxidative stress mouse model. This provided a platform for researching protein-mediated stress responses that impact fertility by revealing changes in antioxidant enzyme activity (CAT, SOD, and GSH-Px) as well as structural ovarian alterations. In the study of Satrio et al. [88], age had a significant impact on the sperm proteome of Simmental bulls, where younger bulls expressed proteins linked to acrosome assembly and spermatid development (SPACA1), middle-aged bulls exhibited motility and decapacitation markers (PEBP1), and older bulls exhibited proteins linked to capacitation-associated hyperactivity. Figure 7, below, shows the application of proteomics techniques described in Figure 6, analysing Toraya buffalo sperm and seminal plasma. First, an artificial vagina was used to extract the sample (semen) from the animal (Toraya buffalo). The sample was then divided into sperm cells and seminal plasma. The seminal plasma and sperm cells were then used to extract the proteins, and the concentrations of those proteins were subsequently analysed. Thereafter, these proteins were separated by their molecular weight on a gel using the One-Dimensional SDS-PAGE technique. Enzymes such as trypsin break down the trapped proteins into smaller fragments, known as peptides. The peptides are then dried in a vacuum concentrator to prepare them for extremely sensitive analysis using nano-LC-MS/MS. This approach determines each peptide’s precise mass and sequence. Thereafter, the raw extract is analysed using a variety of software databases, such as UniProt, STRING, and PANTHER, to determine which particular proteins were present and to comprehend their biological roles and interactions.

#### 3.2.4. Metabolomics

Metabolomics technology has been used to define the association between animal reproduction performance and metabolic changes to gain a better understanding of the factors influencing reproductive results. This technology has characterised information on the utilisation of nutrients, energy metabolism, and biomarkers in reproductive activities of animals [90]. Indeed, this is a useful tool for studying animal reproduction because it makes it possible to examine the metabolic alterations that occur during various reproduction stages, including ovulation, follicular development, spermatogenesis, fertilisation, and embryogenesis [91]. The use of metabolomics technology has helped characterise the relationship between animal reproduction performance and metabolic alterations and further identify the variables that influence the reproductive outcomes [92]. It also identifies the unique metabolic indicators associated with fertility by analysing the metabolite profiles of various reproductive organs, fluids, and biofluids [93].

There are two types of metabolomics: targeted and non-targeted. Targeted metabolomics provides quantitative data for target metabolites that are part of metabolic pathways, addressing specific biological hypotheses. This critical metabolomics technique used in animal reproduction research quantifies the volume of special metabolites included in hormone synthesis and embryonic growth [94]. Non-targeted metabolomics generates hypotheses used to identify targets from the beginning by characterising as many metabolites as possible in biological samples without any prior information about their chemical identification. They can also be utilised to identify new metabolomic pathways or biomarkers associated with animal reproduction [95].

**Figure 8:**
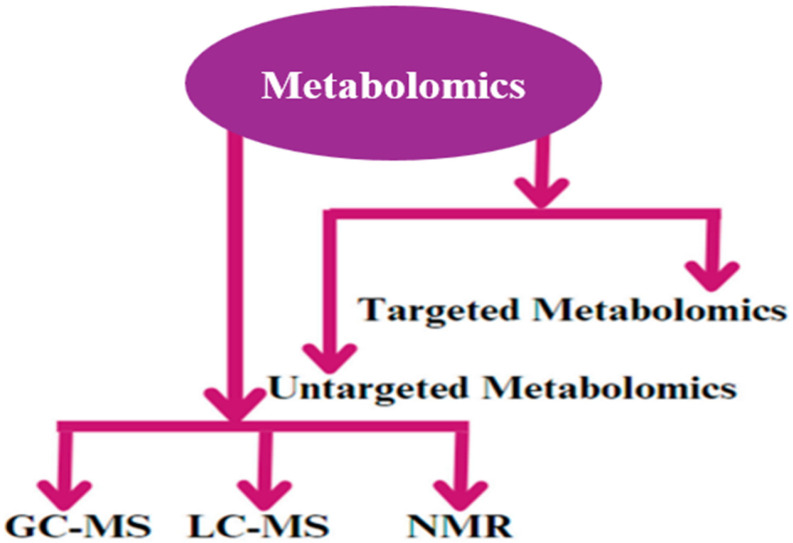
Various types of metabolomics techniques used in biological sample analysis (source: [46]).

**Figure 9:**
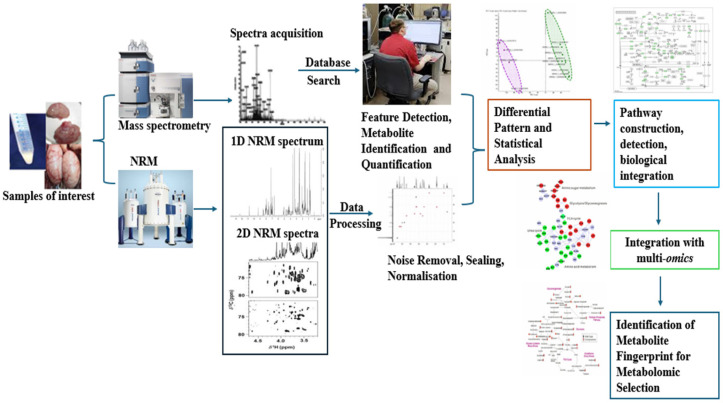
Workflow for application of metabolomics on genetic selection of animals using gas chromatography–mass spectrometry (GC-MS), liquid chromatography–mass spectrometry (LC-MS), and nuclear magnetic resonance (NMR) spectrometry techniques [62].

It has been shown that metabolites play a significant role in maintaining cellular homeostasis by acting as biological modulators across multilayer *omics* and, unlike genomes, transcriptomes, proteomes, and metals, can be gathered in a cell that contains all biomolecules, known as a metabolome [15]. The profiling of metabolomes has been used to identify candidate genes and metabolites, as well as to reveal metabolic mechanisms of therapeutic efficacy [96]. Many techniques, such as Fourier transform infrared (FT-IR) spectroscopy, Raman spectroscopy, nuclear magnetic resonance (NMR) spectroscopy, and MS-based techniques like MS, MS/MS, liquid chromatography (LC)-MS, and gas chromatography (GC)-MS, have been developed to characterise the metabolome. The most practical technique for probing the metabolome and detecting a wide variety of metabolites among these approaches is MS [97]. Figure 8, below, depicts various techniques used in metabolomics analysis of chosen samples, which is further depicted in a practical workflow in Figure 9, where the various techniques, including LC-MS and NRM, are applied to the selected biological sample for the genetic selection of animals.

### 3.3. Integration of High-Throughput Omics Technologies with ARTs

Each of the aforementioned *omics* technologies can be used in the field of assisted reproduction to identify the ideal molecular characteristics of the cells and tissues involved in reproduction, such as spermatozoa, testis, oocytes, embryos, and their metabolic products, such as seminal plasma, follicular fluid, and culture media [98]. The success of assisted reproduction can be increased by using this system biology method to identify the best spermatozoa and oocyte for fertilisation and the best embryo for implantation and live birth [99]. Analysing these cells is therefore essential since they are crucial to reproduction. ARTs have since been used in animal production with the goal of accelerating genetics and improving reproductive efficiency. This covers technologies including somatic cell nuclear transfer, in vitro fertilisation, artificial insemination, and embryo transfer [10]. Their integration with *omics* technologies like genomics, transcriptomics, proteomics, and metabolomics in animal biotechnology offers the potential to significantly enhance reproductive outputs in farm animal production through the provision of a deeper understanding of the molecular physiological mechanisms behind fertility [100]. This integration aims to improve in vitro fertilisation success rates and identify causes of infertility by going beyond the conventional morphology-based selection of embryos to thorough molecular profiling [88]. Additionally, integrating data from different *omics* technologies can uncover intricate biochemical pathways and regulatory networks that underlie important characteristics. However, this necessitates rigorous statistical analysis and the careful consideration of data compatibility [66]. To fully utilise *omics* technologies in animal biotechnology, computational models and machine learning techniques must be developed [88].

These techniques make it simpler to identify bulls with low fertility, but it is more difficult to rank those with moderate-to-high fertility [97]. To determine the appropriate spermatic parameters for semen fertility prediction, several studies, such as those by Sellem et al. [101], Gliozzi et al. [102], and Kumaresan et al. [103], combined multiple in vitro evaluations and obtained an accuracy of R^2^ = 0.39, R^2^ = 0.84, and R^2^ = 0.83, respectively, for the developed model for male fertility prediction in cattle species. These investigations have shown that even in the high-fertility range, bulls’ fertility levels could be distinguished using a combination of kinetic semen parameters and DNA analysis based on flow cytometry, such as DNA sperm integrity.

Turri et al. [104] emphasised in their work that semen performance analysis using conventional methods does not provide a sufficient description of the male fertility level. Therefore, to offer a definitive answer to this issue, more intricate and advanced technological techniques are required to identify male fertility. Transcriptome analysis is one such technique. Özbek et al. [105] further supported this by emphasising in their study that *omics* technology, specifically transcriptome analysis, comprehensively and extremely sensitively screens the molecular dynamics of reproductive indicators at both the cellular and molecular levels. Pig oocytes matured in vitro or in vivo were subjected to transcriptome analysis, and it was shown that the wingless-type MMTV integration site (WNT) signalling components were over-represented in the in vitro-matured oocytes. Dickkopf-related protein 1 (DKK1), a WNT signalling antagonist, was added to the in vitro maturation medium to block the WNT pathway, increasing the number of cells in blastocysts and improving oocyte maturation rates [106]. By analysing short RNA profiles in conjunction with semen quality, Turri et al. [107] showed that a deeper comprehension of sperm function could predict male fertility.

Using genome and transcriptome insights, ARTs can significantly increase animal fertility and reproduction rates. Animals with superior reproductive features, such as early puberty, greater conception rates, and shorter calving intervals, are made available through genomic selection [108]. Therefore, while transcriptomics provides valuable information about the dynamics of gene expression during critical stages of reproduction, such as oocyte maturation, embryo development, and implantation, genome-wide associated studies have identified loci associated with fertility traits, enabling the selection of animals with the necessary genetics [109]. The fundamental molecular pathways of uterine receptivity, embryo implantation, and ART efficacy are thus revealed by its profiling of reproductive tissues [108].

### 3.4. Challenges Associated with the Use of Omics Technologies in Livestock Reproduction

Although integrating *omics* technology with assisted reproductive technologies (ARTs) to enhance farm animals’ reproductive efficiency has some positive results, there are various challenges that need to be addressed. Primarily, *omics* technologies and their integration with ATRs produce vast amounts of complicated data, mostly gigabytes to terabytes, often referred to as “Big Data”, which is difficult for conventional management solutions to handle [110]. Therefore, expert bioinformaticians, statisticians, and computer scientists are needed to critically evaluate, analyse, and interpret this dataset. In order to prevent erroneous results and misinterpretation of research data, sophisticated statistical approaches and models must be implemented. Typically, this enormous amount of high-dimensional data originates from the simultaneous evaluations of genes, transcripts, proteins, and metabolites [111]. Furthermore, the high cost of *omics* technologies poses a significant obstacle to their regular use on a larger scale and makes them less affordable at the farm level. To save expenses, the technology currently remains in the lab until point-of-care devices, such as a dipstick test for complicated metabolites, are developed. Additionally, it is a huge step to transfer *omics*-based biomarkers from a controlled laboratory setting to the real farm. Although this is excellent at spotting possible characteristics, there are still several practical obstacles that prevent the translation gap from closing.

Another constraint is associated with the polygenic genetic architecture of fertility traits, which are often altered by intricate interactions between genetic and environmental factors. Their polygenic nature makes it difficult to identify genetic variants associated with fertility, because these variables are typically influenced by numerous small-effect loci rather than a single, easily identifiable gene [112]. Longitudinal studies are difficult in animals with low reproductive rates and lengthy generation intervals, like cattle, because of the limited generational turnover, which makes it difficult to locate and validate fertility-related loci [113]. To advance the application of *omics*-driven insights to enhance livestock reproductive efficiency, these issues must be resolved.

### 3.5. Prospects for the Continuous Use of High-Throughput Omics Technologies in Animal Reproduction

The cost of some of the more sophisticated technologies is still a problem; however, data integration and increased efficiency are meant to solve this. These high-throughput *omics* technologies offer a novel analytical method that enables a whole biological readout with the goal of enhancing farm animals’ reproductive capabilities through analysing thousands of molecules simultaneously [114]. This is great and promotes a shift to precision breeding for better health, fertility, and efficiency by uncovering the molecular underpinnings of traits like disease resistance, improving genetic selection (genomics), predicting reproductive success (proteomics), and integrating with ARTs for sustainable livestock systems [115]. But building a large animal database is challenging. By so doing, these methods have the potential to greatly improve our knowledge of the crucial mechanisms governing animal reproduction in general and fertility in particular.

It has therefore been demonstrated that integrating *omics* technologies such as transcriptomics, proteomics, metabolomics, and genomics provides a comprehensive understanding of reproductive processes and reveals regulatory networks and new biomarkers for intervention and breeding selection [116]. This could offer a precision-oriented, data-driven method for evaluating production with ecological viability, thereby setting a new benchmark for farm animal management worldwide. By studying sperm-specific proteins, the integration of *omics* with ARTs can effectively improve semen sorting and develop technologies such as embryo transfer. Precision in reproductive management and more sustainable and effective animal production systems will continue to benefit from these advancements [117].

Global food security and the environment could be greatly impacted by the widespread use of *omics* technologies in animal farming. Their role in farm animals’ reproduction is constantly evolving and expanding. These approaches provide avenues to more resilient and sustainable animal production systems by improving reproductive efficacy and reducing the ecological impact of livestock farming, addressing the expanding food targets in an environmentally appropriate way [10]. Therefore, it is essential to make ongoing investments in research and development utilising these cutting-edge technologies to fully exploit their promise in the reproductive biology of farm animals.

## 4. Conclusions

Commonly used assisted reproductive technologies (ARTs) have been used for years and have reached their peak efficiency. Hence, their integration with high-throughput technologies, such as genomics, transcriptomics, proteomics, and metabolomics, has become critical for advancing animal reproductive performance. By providing unprecedented insights into complex biological processes, these tools have transitioned from discovery phase research to practical applications in fertility preservation and molecular breeding. However, the resulting “Big Data” necessitates specialised bioinformatics and computational expertise to translate complex datasets into actionable breeding strategies. Despite their potential, further research is required to fully bridge the gap between *omics* data and field-level implementation. Future efforts should prioritise the integration of multi-*omics* with ARTs to enhance the precision and sustainability of livestock systems. Ultimately, refining these high-throughput applications will be critical for improving reproductive efficiency, reducing the environmental footprint of farming, and securing global food security.

## Figures and Tables

**Table 1:** Grouping of published articles according to period of publication and use of high-throughput *omics* technologies in farm animals’ reproductive biology.

*Omics*	Period of Publication			Total
	2010–2015	2016–2020	2021–2025	
Genomics	3	5	2	10
Transcriptomics	2	2	4	8
Proteomics	0	1	1	2
Metabolomics	2	2	3	7
Grand total				27

## Data Availability

No new data were created or analyzed in this study. Data sharing is not applicable to this article.
